# Cannons and sparrows: an exact maximum likelihood non-parametric test for meta-analysis of k 2 × 2 tables

**DOI:** 10.1186/s12982-018-0077-7

**Published:** 2018-06-26

**Authors:** Lawrence M. Paul

**Affiliations:** Somerset, NJ USA

**Keywords:** Meta-analysis, Categorical analysis, Mantel–Haenszel, DerSimonian–Laird, Exact solution, Inverse variance

## Abstract

**Background:**

The use of meta-analysis to aggregate multiple studies has increased dramatically over the last 30 years. For meta-analysis of homogeneous data where the effect sizes for the studies contributing to the meta-analysis differ only by statistical error, the Mantel–Haenszel technique has typically been utilized. If homogeneity cannot be assumed or established, the most popular technique is the inverse-variance DerSimonian–Laird technique. However, both of these techniques are based on large sample, asymptotic assumptions and are, at best, an approximation especially when the number of cases observed in any cell of the corresponding contingency tables is small.

**Results:**

This paper develops an exact, non-parametric test based on a maximum likelihood test statistic as an alternative to the asymptotic techniques. Further, the test can be used across a wide range of heterogeneity. Monte Carlo simulations show that for the homogeneous case, the ML-NP-EXACT technique to be generally more powerful than the DerSimonian–Laird inverse-variance technique for realistic, smaller values of disease probability, and across a large range of odds ratios, number of contributing studies, and sample size. Possibly most important, for large values of heterogeneity, the pre-specified level of Type I Error is much better maintained by the ML-NP-EXACT technique relative to the DerSimonian–Laird technique. A fully tested implementation in the R statistical language is freely available from the author.

**Conclusions:**

This research has developed an exact test for the meta-analysis of dichotomous data. The ML-NP-EXACT technique was strongly superior to the DerSimonian–Laird technique in maintaining a pre-specified level of Type I Error. As shown, the DerSimonian–Laird technique demonstrated many large violations of this level. Given the various biases towards finding statistical significance prevalent in epidemiology today, a strong focus on maintaining a pre-specified level of Type I Error would seem critical.

## Background

**Fig. 1:**
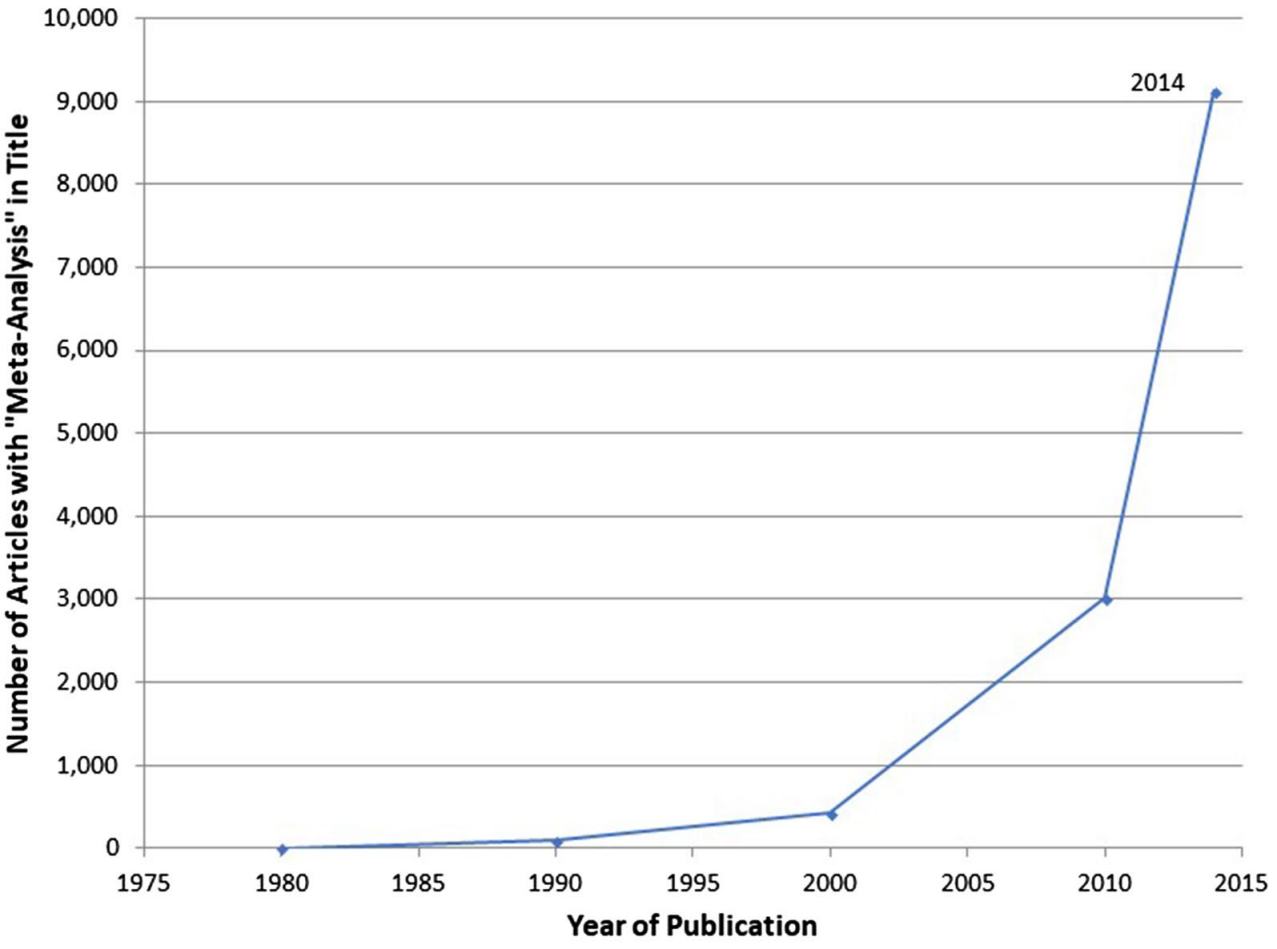
Number of articles containing “meta-analysis” in the title by year of publication

The use of meta-analysis in epidemiological research has been increasing at a very rapid rate. A review of the National Library of Medicine’s online database (“Pub Med”) shows that in 1980 there were only five research articles with the phrase “meta-analysis” in their titles. The number had increased to 92 in 1990, 422 in 2000, and to 9125 in 2014 (see Fig. 1).

While part of this growth may be due to the widespread availability of powerful personal computer software making meta-analysis techniques easier to perform and more feasible to implement, this growth also likely represents a critical epidemiological need to draw meaningful conclusions from an aggregation of studies.

The use of meta-analytic techniques is controversial when the contributing studies are not randomized control trials (“RCT”). Many researchers feel that it is highly misleading to attempt to combine a series of disparate studies [1, 2] while others maintain that, with proper safeguards, meta-analysis allows an extremely useful pooling of smaller studies [3, 4]. A discussion of the appropriateness of meta-analysis is beyond the scope of this paper. Rather, the focus here will be on minimizing unnecessary error in testing the overall statistical significance of a meta-analysis.

### Overview of 2 × 2 × k categorical meta-analysis

The “2 × 2 × k” categorical meta-analysis paradigm is probably the most frequently encountered situation in meta-analysis. It consists of a series of *k* contributing studies each described by a 2 × 2 contingency table. Each cell of the 2 × 2 table contains the number of occurrences of an event (e.g., a disease case) for the particular combination of row and column variables. For the sake of exposition, we can associate the two columns of each table with “Disease Manifestation” versus “No Disease Manifestation” and the two rows with “Exposure” versus “No Exposure.” Table 1 represents the results of one of the *k* studies

**Table 1:** Typical contributing study (one of *k*) in a dichotomous meta-analysis

Treatment	Disease status		
	Disease manifestation	No disease manifestation	Total
Exposure	*r*_*k*_ = 4	96	100
No exposure	*s*_*k*_ = 2	98	100
Total	*t*_*k*_ = 6	194	200

In most meta-analyses, there are typically two distinct components: (1) a statistical test of the overall difference between the “Exposure” and “No Exposure” groups across the *k* contributing studies, and (2) a method to pool the observed differences between groups across the *k* studies in order to estimate the true difference (the “Effect Size”).

Many epidemiologists employing meta-analytic techniques have greatly deemphasized the first component in recent years. Borenstein et al. [3] conclude:

“… However, meta-analysis also allows us to move beyond the question of statistical significance, and address questions that are more interesting and also more relevant.” (pp. 11–12).

Similarly, Higgins and Green [4] rather dismissively state:

“… If review authors decide to present a P value with the results of a meta-analysis, they should report a precise P value, together with the 95% confidence interval” (pp. 371–372).

This study addresses only the first of these two components. A method is developed that attempts to maintain the Type I error (“false alarm rate”) at the desired level but has good power to detect true differences across a large range of event probability, number of contributing studies, sample size and level of heterogeneity.

An argument can be made that maintaining the Type I error at a pre-specified level is more important than the power (1—Type II error rate) to detect true differences between conditions. The framers of modern statistical testing called such errors “Errors of the First Kind” and placed a special emphasis on them. Neyman & Pearson in 1933 stated:

“A new basis has been introduced for choosing among criteria available for testing any given statistical hypothesis, H_*0*_, with regard to an alternative H_*t*_. If ϴ_*1*_ and ϴ_*2*_ are two such possible criteria and if in using them there is the same chance, ε, of rejecting H_*0*_ when it is in fact true, we should choose that one of the two which assures the minimum chance of accepting H_*0*_ when the true hypothesis is H_*t*_.” [5] [p. 336]

Thus, while Neyman and Pearson supported the effort to choose criteria that yield the greatest power to detect true differences, this effort is secondary to maintaining a pre-specified level of Type I error. Estimating the Effect Size particularly for rare events is well covered in a number of recent studies (see in particular [6, 7]).

### “Rare” events and meta-analysis

The probability of occurrence of a disease is often categorized as “rare” although no specific definition exists. As an example, Higgins et al. state that “There is no single risk at which events are classified as ‘rare’” but gives as examples 1 in a 1000 or 1 in a 100 (see [8], p. 520). An obvious related issue is observing zero cases in one or more cells of a contingency table. Table 2 shows the expected cell sizes from various realistic combinations of disease probability and individual study sample size.

**Table 2:** Expected number of disease cases in each study arm as a function of disease probability and individual study sample size

Disease/condition	Approximate disease probability	Individual study sample size (each arm)		
		100	500	1000
Myocardial infarction	.0025	.25	1.25	2.5
Parkinson’s disease (60–65 age group)	.00039	.039	.195	.39
Alzheimer’s disease (60–65 age group)	.0008	.08	0.4	0.8
Overall cancer for men	.0055	.55	2.75	5.5

If one heuristically defines “rare” as fewer than five expected cases of a disease, Table 2 supports the notion that “rare” events are a focus of many epidemiological studies.

For homogeneous meta-analysis (i.e. where the effect across studies may be assumed to be the same within statistical variation), the two techniques typically used for categorical data are the Mantel–Haenszel and Peto techniques. Both of these techniques rely on the Mantel–Haenszel Chi Square to test for the overall statistical significance. For heterogeneous meta-analyses, the DerSimonian–Laird inverse variance technique (DL) which requires a number of assumptions is typically used [9]. The technique developed in this paper will be compared directly to the DL technique as described below.

The problem in applying large sample asymptotic techniques to meta-analyses involving small numbers of cases will be illustrated in the older and more developed domain of homogeneous meta-analyses. Mantel and Haenszel developed what is probably the most used technique for homogeneous meta-analyses [10]. In applying this technique, Manel and Fleiss showed that a minimum of approximately five cases was required in each of the 4 cells of each of the 2 × 2 tables for each of the *k* studies comprising the meta-analysis [11]. This is the same requirement typically used without any particular justification for the simple Chi square test. All but one of the combinations of individual study sample size and disease probability shown in Table 2 would yield fewer than five cases per cell leading to violations of the minimum cell size in the asymptotic Mantel–Haenszel (MH) Chi Square test, and thus the test would be potentially flawed.

R. A. Fisher addressed the limitations of using asymptotic large sample methods in 1925 in the preface to the first edition of his well-known “Statistical Methods for Research Workers” [12]:

Little experience is sufficient to show that the traditional machinery of statistical processes is wholly unsuited to the needs of practical research. Not only does it take a cannon to shoot a sparrow, but it misses the sparrow. The elaborate mechanism built on the theory of infinitely large samples is not accurate enough for simple laboratory data. Only by systematically tackling small sample problems on their merits does it seem possible to apply accurate tests to practical data.

The continued use asymptotic tests in situations not suited for their use is unacceptable given the computer power that is now available to all researchers.

### Heterogeneity versus homogeneity in meta-analyses

The term “heterogeneity” refers to the fact that studies done at different times and by different researchers might be expected to have different treatment effects. The expectation is that a variable of interest may owe its effect, at least in part, to one or more other variables. The meta-analysis researcher, J. P. T. Higgins stated: “Heterogeneity is to be expected in a meta-analysis: it would be surprising if multiple studies, performed by different teams in different places with different methods, all ended up estimating the same underlying parameter.” ([13], p. 1158). While researchers may agree that heterogeneity is to be expected, there is very little agreement on how to quantify this variability. The most obvious and direct candidate is τ^2^, the assumed variability between studies. However, τ^2^ is not invariant across study designs and its interpretation may not be intuitive. Alternatives include *I*^*2*^, the ratio of the inter-study variability to the total variability and the *Q* statistic, which is mathematically related to *I*^*2*^ (see, e.g., [14]).

In the technique described in this paper, heterogeneity will be mathematically manipulated through τ^2^ and the logit function using the same approach as Bhaumik et al. [15]. Namely,

1 $$\begin{aligned} & x_{ic} \sim B\left( {p_{ic,} n_{ic} } \right), x_{it} \sim B\left( {p_{it,} n_{it} } \right), \hfill \\  {\text{logit}} \left( {p_{ic} } \right) = \mu , {\text{logit}} \left( {p_{it} } \right) = \mu + \theta + \varepsilon_{i} \hfill \\ \varepsilon_{i} \sim N\left( {0,\tau^{2} } \right) \hfill \\ \end{aligned}$$

where *B* is the Binomial Distribution, *N* is the Normal Distribution, *x*_*ic*_*, x*_*it*_ are the observed number of cases in the control and exposure groups respectively of the *i*th study, *p*_*ic*_*, p*_*it*_ are the event probabilities in the control and exposure groups respectively of the *i*th study, *n*_*ic*_*, n*_*it*_ are the sample sizes in the two groups of the *i*th study, *µ* corresponds to the background event probability in the treatment and control groups, $\theta$ corresponds to the overall Odds Ratio for the Exposure Group relative to the Control Group or the “log of the odds ratio”, $\tau^{2}$ is a variance corresponding to the heterogeneity or the “heterogeneity parameter”, $\varepsilon_{i}$ is the deviation in the treatment group of each of the contributing studies due to heterogeneity.

### The basic principles of the DerSimonian–Laird (DL) method

As stated above, this research specifically contrasts an exact method for conducting meta-analyses in *k* 2 × 2 tables with heterogeneity with the most popular approach which was developed by DerSimonian and Laird [9] (DL).

For each contributing study, the DL technique calculates the logarithm of the sample odds ratio and a corresponding estimate of the variance of this measure based on the asymptotic distribution of these logarithms. Adjustments are made for entries in the individual 2 × 2 tables that contain a zero-cell count. Equations 2–5 below capture the core DL approach. In Eq. 2, an estimate of the interstudy variability, $\tau^{2}$, is first derived from Cochran’s Q statistic and the weights assigned to each of the *k* contributing studies, $\omega_{i}$. These weights are equal to the inverse of the square of the standard error of the estimate of the odds ratio, $\hat{\theta }_{i } ,$ in each of the *k* contributing studies.

2 $$\hat{\tau }^{2} = \frac{{Q - \left( {k - 1} \right)}}{{\sum \omega_{i} - \left( {\frac{{\sum \omega_{i}^{2} }}{{\sum \omega_{i} }}} \right)}}$$

As shown in Eq. 3, a new set of weights, $\omega_{i}^{{\prime }}$, are then calculated based on the estimated value of $\hat{\tau }^{2}$ from Eq. 2 and the standard errors of the contributing studies.

3 $$\omega_{i}^{{\prime }} = \frac{1}{{SE\left( {\hat{\theta }_{i } } \right)^{2} + \tau^{2} }}$$

These new weights are then used to calculate estimates of both the overall log odds ratio, $\theta_{DL }$ and its standard error as shown in Eq. 4 and 5.

4 $$\hat{\theta }_{DL } = \frac{{\sum \omega_{i}^{{\prime }} \hat{\theta }_{i } }}{{\sum \omega_{i}^{{\prime }} }}$$

5 $$SE\left( {\hat{\theta }_{DL} } \right) = \frac{1}{{\sqrt {\sum \omega_{i}^{{\prime }} } }}$$

A test of statistical significance is then based on a large sample normal distribution. The DL technique requires asymptotic assumptions regarding both the Q statistic used to estimate the interstudy variability, $\tau^{2} ,$ and the normal distribution required to test for statistical significance. A more subtle issue is the possibility of distorting correlations between the individual estimates of the effect size for each contributing study, $\theta_{i }$, and the individual weights used for each of these contributing effect sizes.

### The ML-NP-EXACT: an exact maximum likelihood non-parametric test of 2 × 2 × k dichotomous data

#### Basic approach

An exact approach to developing a maximum likelihood test of independence for k 2 × 2 tables logically starts by first addressing the simple *k *= 1 2 × 2 table case. An exact method would use maximum likelihood estimates of the cell counts and associated cell probabilities and then use a “goodness of fit” test sensitive to violations of independence. Agresti and Wackerly [16] argued that “exact conditional tests can be simply formulated by using other criteria for ranking the tables according to the deviation each exhibits from independence.” [pp. 113–114] and go on to mention likelihood ratio statistics.

One such statistic is the *G Test* “goodness of fit” statistic strongly advocated by Sokal and Rohlf [17] to test for independence between the row and column variables. Sokal and Rohlf cite Kullback and Leibler’s “Divergence” measure which is mathematically identical to the *G* Test [18]. The probability distribution of the *G Test* statistic is asymptotically χ^2^ which would be adequate for tables with large numbers in each of the cells. However, for the case of sparse tables being developed in this paper, this would not be satisfactory. For this simple *k *= 1 case, Fisher’s Exact Test would be appropriate [19]. Fisher’s Exact Test exploits the fact that if one conditions on any of the marginal totals, the cell frequency of interest will be a sufficient statistic. Then, the associated frequency distribution of the cell frequency of interest may be determined exactly using the hypergeometric distribution. Fisher’s Exact approach has been extended by Thomas [20] and others. Among other advantages, such conditioning eliminates the effect of any nuisance variable identically affecting both exposure categories. Using Table 1 nomenclature, the number of individuals manifesting the disease being studied in the Exposure Group, *r*_*k*_, conditionalized on the total number of individuals manifesting the disease, *t*_*k*_ is a sufficient statistic. This approach can be directly extended to 2 × 2 × *k* designs by again using the *G Test* “goodness of fit” statistic and testing for conditional independence in each of the *k* tables comprising the overall meta-analysis.

In the 3-way 2 × 2 × *k* meta-analysis, one approach is to first test for independence among the two factors (Disease Status and Treatment in the terminology of Table 1) in each of the *k* strata (e.g. using the Breslow-Day test of interaction [21]). If such a test of interaction supported independence of the two factors, the notion of a Common Odds Ratio (COR) could be entertained. Then the overall COR averaged across the *k* strata could be tested against the null hypothesis of 1.0 using the Cochran-Mantel–Haenszel test or equivalent.

Alternatively, Yao and Tritchler [22] developed an exact conditional independence test for 2 × 2 × *k* categorical data. Although they derived an exact null hypothesis frequency distribution, they chose to use the standard Chi Square test statistic:

6 $$\chi^{2} = \chi_{1}^{2} + \chi_{2}^{2} + \cdots \chi_{k}^{2}$$

where *k* is the number of contributing studies.

 The present author programmed their test in the R statistical language based on Yao’s dissertation [23]. Preliminary Monte Carlo simulations showed, however, that this implementation yielded a test with limited power compared to the DL method. With the advantage of the hindsight provided by this simulation, the use of a Chi Square statistic for this exact test is probably suboptimal and is not necessary given their derivation of an exact null hypothesis frequency distribution.

A straightforward utilization of *G Test* per [17] would thus be:

$$G^{2} = 2\mathop \sum \limits_{i = 1}^{k} O_{i } \ln \left( {\frac{{O_{i } }}{{E_{i } }}} \right)$$

where *k* is the number of contributing studies, *O*_*i*_ is the number of observed cases in the Exposure Group of the *i*th contributing study, *E*_*i*_ is the number of expected cases in the Exposure Group of the *i*th contributing study assuming conditional independence.

Table 1 which shows the data for a particular one of the *k* contributing studies will be used to help clarify this approach. There are two sources of cases, cases from the “Exposure” group and cases from the “No Exposure” group. The number of observed cases in the Exposure Group (*r*_*k*_ of Table 1) per Eq. 3 is 4. The number of expected cases under Fisher’s conditional independence approach would be the total number of cases of 6 multiplied by the proportion of the overall sample size corresponding to the Exposure Group which in this case would be .5. Thus, the number of expected cases in the Exposure Group would be 6 *.5 = 3 cases.

The approach being developed in this paper attempts to deal with “rare” events including the possibility of no disease events in either the Exposure group or in the No Exposure group. However, when the number of cases in the Exposure Group, *O*_*i*_, is zero, the $\ln \frac{{O_{i} }}{{E_{i} }}$ term would not be calculable. Simply eliminating such studies would likely lead to an anti-conservative bias in Type I Error. Thus, the following modified *G Test* statistic was used and will be referred to as G^*^:

$$G^{*} = 2\mathop \sum \limits_{i = 1}^{k} \left( {O_{i } + 1} \right)\ln \left( {\frac{{O_{i } + 1}}{{E_{i } + 1}}} \right)$$

This transformation permits calculation of the test statistic when the number of cases in one of the two groups equals zero, but where there are a positive number of cases in the other group. This issue and a similar approach of adding a constant to both the number of Observed and Expected cases was more fully explored in a recent Ph.D. dissertation [24].

#### Two special cases under large heterogeneity

Protocols were developed to handle two special situations under large heterogeneity. The first situation involves the event probabilities in the control and exposure groups respectively of the *i*th study, *p*_*ic,*_ *p*_*it*_, as originally presented in Eq. 1 and shown for convenience below:

$${\text{logit}} \left( {p_{ic} } \right) = \mu , {\text{logit}} \left( {p_{it} } \right) = \mu + \theta + \varepsilon_{i}$$

**Fig. 2:**
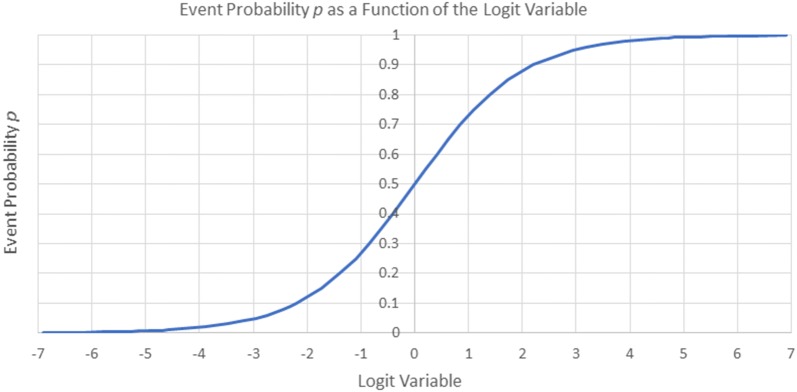
Plot of event probability, *p,* as a function of the logit variable

As shown in Fig. 2 plotted with *p* as a function of the logit variable, *p* has a slope of exactly .25 at *p * = * .5* with the slope approaching zero as *p* approaches both zero and 1.0. In addition, the curve is only symmetric in the logit variable at exactly *p *=.5.

For realistic values of event probability such as .01, positive event probability excursions will be much larger than negative ones for large values of heterogeneity across studies. This problem manifests itself in artificially large violations of the pre-specified level of Type I Error. Therefore, negative values of the test statistic $G$ which corresponds to observing fewer cases in the Exposure Group than expected were increased in magnitude (made more negative) by multiplying them by a correction factor based on the derivative of *p* with respect to the logit variable:

$$Correction \;Factor_{\text{logit}} = \frac{.25}{{\left( {\frac{{dp\left( {\text{logit}} \right)}}{d\text{logit}}} \right)}} = \frac{.25}{{\frac{{\exp \left( {\text{logit}} \right)}}{{\left\{ {1 + \exp \left( {\text{logit}} \right)} \right\}^{2} }}}}$$

As required, the correction factor equals one when *p * = .5 and becomes appropriately large as *p* approaches zero. This correction factor was applied when the $G^{*}$ statistic was negative and when the treatment variance relative to the control variance was greater than or equal to 3.0.

A second problem concerns meta-analyses in which there are only a small number of contributing studies. As shown by InHout and colleagues [25], there is a monotonic and large positive effect on Type I Error as the number of contributing studies decreases. This is not a problem of inadequate replications, but one of bias. The author conducted a separate analysis that showed that under large heterogeneity there is a corresponding large probability for a single study to incorrectly skew the overall test statistic towards statistical significance. When the number of contributing studies is large, this tendency is counterbalanced by other studies having correspondingly large excursions in the negative direction.

**Fig. 3:**
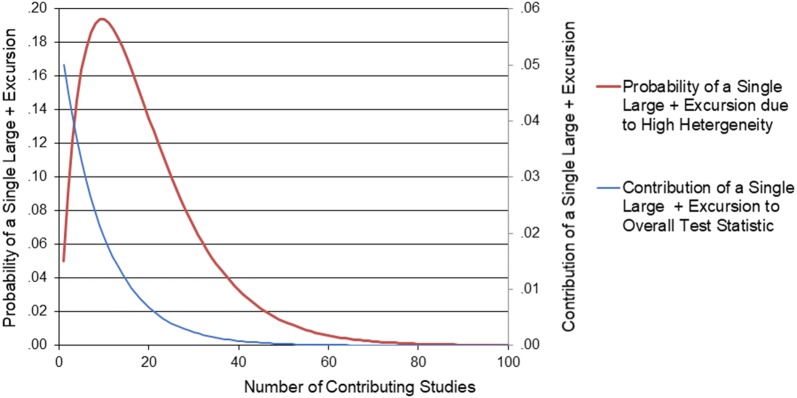
Plot of probability of a single “skewing” event and its contribution to the overall test statistic as a function of the number of contributing studies

This problem can be appreciated in Fig. 3.

The first curve plotted on the primary vertical axis is the probability of obtaining a single large positive excursion likely due to a high level of heterogeneity in any one of the *k* contributing studies. The second curve plotted on the secondary vertical axis is simply the first variable divided by the number of studies on the x axis. This second variable is thus proportional to the contribution made by this single study’s large positive excursion to the average test statistic. As the plot of this second variable suggests, the contribution of a single large positive excursion study is monotonically decreasing with increasing number of contributing studies as the effect of averaging begins to be exhibited. The correction algorithm that was used identified those situations when the overall test statistic was greater than the critical value and .75 or more of the test statistic came from a single large positive excursion. When this situation obtained, the contribution of this likely outlier to the overall test statistics was eliminated.

### Implementation of the test in the R statistical language

This conditional-independence maximum likelihood exact test was implemented in the R Statistical Language. Each of the *k* contributing studies has a discrete probability distribution which is a function of the background event probability and odds ratio of that study. The joint probability distribution of the *k* discrete probability distributions is the convolution of these *k* distributions which will also be a discrete distribution. Directly performing this convolution would be extremely time consuming even for relatively small values of *k.* However, it can be readily shown that if each of the *k* distributions is first transformed into the “frequency domain” using the Fourier Transform, the simple multiplicative product of these *k* transformations is the Fourier transform of the joint probability distribution. A single inverse Fourier transform then yields the joint probability distribution (see, e.g., [26]). This was the approach used by Yao & Tritchler [22]. The development of the Fast Fourier Transform (FFT) for generation of the Fourier Transform in discrete situations [see, e.g. [27] for a relatively early presentation] such as the categorical meta-analysis presented here permits quickly determining the joint probability distribution using almost any PC-type computer. The program has been extensively tested across a large range of heterogeneity, odds ratios, disease probability, number of contributing studies and sample size.

### Monte Carlo simulation of the ML-NP-EXACT and DerSimonian–Laird techniques

#### Population-based odds ratio simulation

A series of Monte Carlo simulations was conducted to evaluate the ML-NP-EXACT technique and compare it directly to the typically used DerSimonian–Laird Inverse Variance technique [9] for population-based odds ratio scenarios. The simulation was written and executed in “R: A Programming Environment for Data Analysis and Graphics.” [28]. The DerSimonian–Laird results were calculated using the “meta” package in R [29].

Five levels of odds ratio were chosen (1.0, 1.25, 1.5, 1.75, and 2.0) which were crossed with three levels of background event probability (.005, .01, and .05), and three levels of sample size (50,100 and 200) in each arm of each contributing study. Finally, the number of studies entering into each meta-analysis was chosen to be 5, 10, 20, or 40 studies.

In addition, the heterogeneity between the contributing studies, τ^2^, was evaluated at 0 (homogeneity), .4, and .8. This last value of .8 represents a very large variance among the studies and was partially chosen to be able to compare the results with previous work [15]. To put such a large inter-study variance into some perspective, a background event probability (e.g. disease probability) of .01 would be expected to fluctuate between .0017 and .057, a ratio of 33:1 under a heterogeneity of τ^2^ = .8.

Finally, the common variability in both the exposure and control groups was chosen to be .5 and an error term, $\varepsilon_{i} = N\left( {0,.5} \right)_{{}}$ was added to both the logit transformed probabilities of Eq. 1 above per Bhaumik*’*s [15] desire to “imply that both the control and treatment groups have varying rates of events” (p. 9) allowing direct comparisons to be made to this earlier research. For each simulation, the overall treatment effect was evaluated using both the ML-NP-EXACT and DerSimonian–Laird techniques. All simulation runs were conducted with 2000 replications. A value of .05 was used as the pre-specified level of Type I Error.

**Fig. 4:**
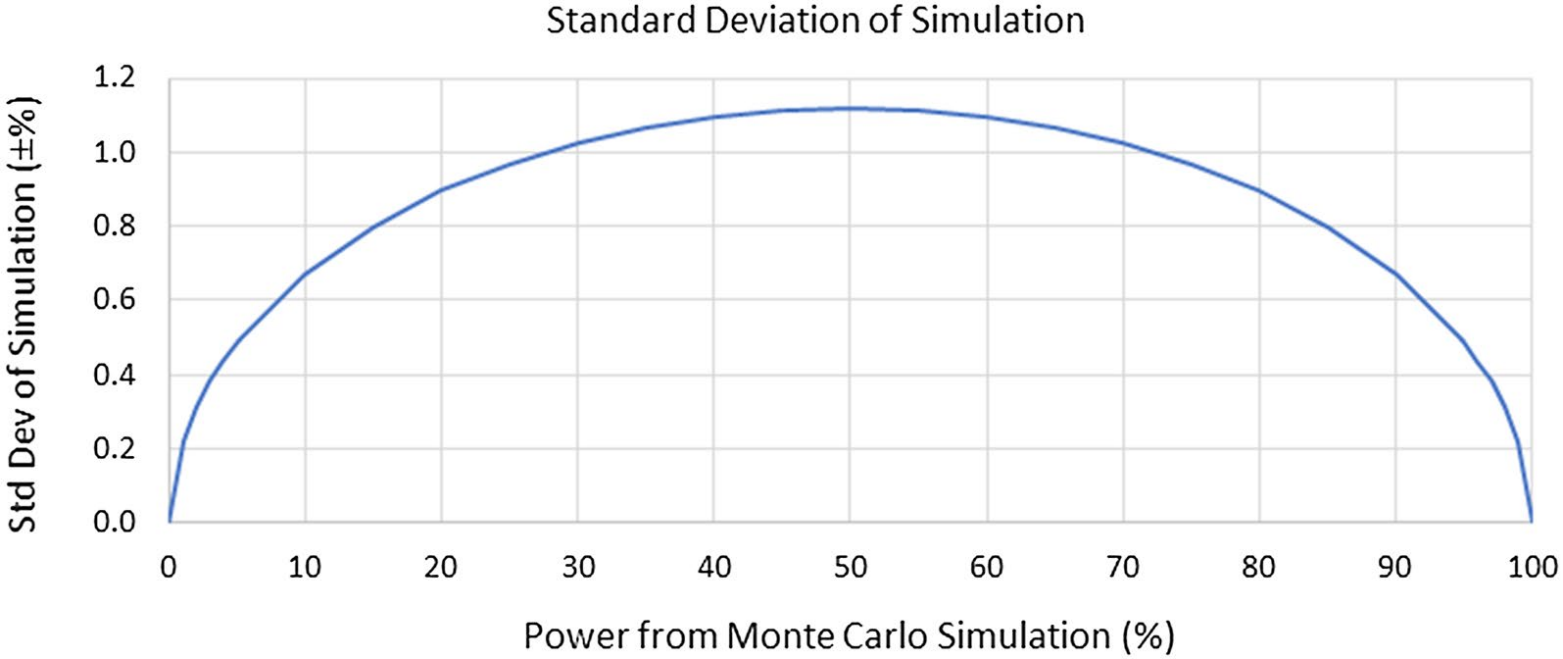
Plot of standard deviation of the simulation as a function of the reported power

The Monte Carlo simulation results are shown below in Tables 3, 4 and 5 corresponding to heterogeneity values *τ*^*2*^ of 0, .4, and .8. respectively. The 108 variable combinations with an Odds Ratio of 1.0 (i.e. no treatment effect) are shown in italics for purposes of exposition. The standard deviation as a function of reported power is shown in Fig. 4.

**Table 3:** Power (%) for the ML-NP-EXACT and DerSimonian–Laird inverse-variance techniques heterogeneity τ^2^ = 0

Row #	Odds ratio	Num. of studies	Sample size	Background event (disease) probability					
				.005		.01		.05	
				ML-NP-EXACT	DerSimon. Inv.-Var.	ML-NP-EXACT	DerSimon. Inv.-Var.	ML-NP-EXACT	DerSimon. Inv.-Var.
1.	*1.0*	*5*	*50*	*6.0*	*0.2*	*4.8*	*0.8*	*5.1*	*7.6*
2.	1.25	5	50	7.9	0.3	6.7	1.8	9.5	8.5
3.	1.5	5	50	8.5	0.2	8.8	2.1	15.8	12.8
4.	1.75	5	50	8.2	0.6	12.6	4.4	21.7	18.0
5.	2.0	5	50	10.7	1.0	15.9	5.4	28.1	23.6
6.	*1.0*	*5*	*100*	*4.4*	*0.5*	*4.7*	*2.8*	*5.7*	*9.0*
7.	1.25	5	100	6.2	1.2	6.9	4.5	12.2	11.9
8.	1.5	5	100	8.2	2.0	10.8	6.3	20.3	16.5
9.	1.75	5	100	12.2	3.9	15.0	10.1	25.3	21.8
10.	2	5	100	14.0	5.4	19.7	15.1	36.7	28.1
11.	*1.0*	*5*	*200*	*4.5*	*3.3*	*5.1*	*5.6*	*7.0*	*11.5*
12.	1.25	5	200	7.6	5.0	9.3	8.5	15.0	13.3
13.	1.5	5	200	9.6	6.0	13.9	12.2	26.7	21.3
14.	1.75	5	200	15.0	8.3	19.8	16.7	38.2	29.4
15.	2	5	200	18.4	12.8	23.1	21.8	46.6	36.8
16.	*1.0*	*10*	*50*	*4.0*	*0.3*	*4.8*	*1.1*	*5.3*	*7.1*
17.	1.25	10	50	6.0	0.5	8.7	2.3	11.0	8.8
18.	1.5	10	50	8.7	1.2	11.8	3.9	22.2	18.5
19.	1.75	10	50	12.8	1.7	17.6	8.0	31.9	25.8
20.	2.0	10	50	14.6	3.6	21.2	11.6	44.0	39.4
21.	*1.0*	*10*	*100*	*3.9*	*1.2*	*4.7*	*2.4*	*5.5*	*7.0*
22.	1.25	10	100	6.8	1.7	7.6	5.1	14.5	11.4
23.	1.5	10	100	11.3	4.0	13.6	8.4	25.8	22.8
24.	1.75	10	100	18.6	7.5	21.5	16.1	40.5	36.1
25.	2	10	100	21.4	11.5	28.2	24.9	52.2	47.7
26.	*1.0*	*10*	*200*	*3.5*	*2.6*	*4.0*	*6.1*	*7.2*	*10.4*
27.	1.25	10	200	9.1	5.4	10.2	8.6	15.4	13.2
28.	1.5	10	200	15.7	9.6	16.1	13.9	29.3	26.6
29.	1.75	10	200	22.7	17.4	27.4	25.5	44.0	40.8
30.	2	10	200	27.8	23.4	37.0	36.9	57.5	56.2
31.	*1.0*	*20*	*50*	*3.5*	*0.7*	*3.8*	*1.0*	*4.5*	*4.8*
32.	1.25	20	50	6.3	1.0	8.9	1.9	14.5	11.4
33.	1.5	20	50	13.1	2.8	17.0	7.1	29.0	27.5
34.	1.75	20	50	17.6	4.8	23.9	14.9	48.1	47.0
35.	2.0	20	50	24.7	9.7	31.7	23.0	62.2	64.0
36.	*1.0*	*20*	*100*	*3.8*	*1.1*	*4.5*	*3.8*	*4.8*	*6.8*
37.	1.25	20	100	8.2	2.5	10.4	6.8	16.8	15.4
38.	1.5	20	100	16.8	6.9	18.9	15.5	35.6	34.7
39.	1.75	20	100	26.0	15.3	31.2	31.5	53.4	57.3
40.	2	20	100	31.5	23.8	40.4	45.1	69.8	75.8
41.	*1.0*	*20*	*200*	*3.6*	*3.6*	*3.8*	*4.6*	*4.6*	*6.7*
42.	1.25	20	200	9.6	5.9	11.8	11.7	16.6	17.6
43.	1.5	20	200	18.8	17.1	24.2	25.2	39.2	42.9
44.	1.75	20	200	28.2	31.2	37.6	44.1	59.8	66.3
45.	2	20	200	38.2	44.8	50.8	61.2	75.1	84.3
46.	*1.0*	*40*	*50*	*5.0*	*0.8*	*5.3*	*1.6*	*3.6*	*4.5*
47.	1.25	40	50	12.6	1.6	13.6	3.8	22.0	17.6
48.	1.5	40	50	20.8	5.0	26.5	14.0	49.0	45.9
49.	1.75	40	50	28.9	11.5	37.9	28.2	70.8	75.8
50.	2.0	40	50	37.3	20.7	48.3	48.6	83.4	91.0
51.	*1.0*	*40*	*100*	*4.5*	*1.9*	*3.7*	*2.4*	*3.4*	*5.6*
52.	1.25	40	100	13.6	4.6	14.1	9.6	24.3	22.7
53.	1.5	40	100	24.3	14.0	27.9	29.0	55.3	59.7
54.	1.75	40	100	35.9	29.5	40.9	53.9	77.3	85.9
55.	2	40	100	49.0	47.6	54.7	74.7	87.8	96.5
56.	*1.0*	*40*	*200*	*3.0*	*3.0*	*3.1*	*5.6*	*3.8*	*5.9*
57.	1.25	40	200	10.9	9.9	16.4	17.0	27.0	27.4
58.	1.5	40	200	29.4	29.7	36.3	44.4	58.7	66.9
59.	1.75	40	200	39.9	54.4	54.7	70.2	81.3	91.9
60.	2	40	200	51.9	75.9	65.6	87.8	92.1	98.5

**Table 4:** Power (%) for the ML-NP-EXACT and DerSimonian–Laird inverse-variance techniques heterogeneity τ^2^ = .4

Row #	Odds ratio	Num. of studies	Sample size	Event (disease) probability					
				.005		.01		.05	
				ML-NP-EXACT	DerSimon. Inv.-Var.	ML-NP-EXACT	DerSimon. Inv.-Var.	ML-NP-EXACT	DerSimon. Inv.-Var.
1.	*1.0*	*5*	*50*	*7.1*	*0.4*	*5.0*	*1.6*	*6.1*	*8.7*
2.	1.25	5	50	7.9	0.5	7.6	3.2	10.3	10.9
3.	1.5	5	50	9.0	1.4	9.1	4.7	15.7	15.5
4.	1.75	5	50	9.6	2.0	11.0	7.3	22.3	19.8
5.	2.0	5	50	12.4	2.9	13.3	8.6	29.9	27.3
6.	*1.0*	*5*	*100*	*5.0*	*1.9*	*4.7*	*3.9*	*6.7*	*10.0*
7.	1.25	5	100	7.4	3.1	8.2	6.2	14.4	13.2
8.	1.5	5	100	9.0	4.7	11.3	9.3	20.7	17.3
9.	1.75	5	100	11.8	6.2	13.8	11.9	30.9	25.7
10.	2	5	100	13.5	9.1	19.6	18.5	38.0	30.1
11.	*1.0*	*5*	*200*	*4.0*	*4.1*	*4.8*	*7.1*	*8.8*	*10.5*
12.	1.25	5	200	8.7	7.6	8.3	9.7	18.3	15.6
13.	1.5	5	200	11.2	9.3	13.6	14.3	27.5	21.0
14.	1.75	5	200	14.6	13.6	17.5	17.8	38.3	28.0
15.	2	5	200	18.0	17.9	24.8	24.2	46.2	34.7
16.	*1.0*	*10*	*50*	*4.6*	*0.7*	*5.2*	*2.6*	*5.7*	*7.4*
17.	1.25	10	50	7.5	1.5	7.9	3.7	13.0	12.5
18.	1.5	10	50	11.1	3.0	12.8	7.7	21.1	20.7
19.	1.75	10	50	13.7	5.2	18.2	13.1	28.4	28.6
20.	2.0	10	50	17.8	8.5	22.4	18.2	40.1	39.6
21.	*1.0*	*10*	*100*	*5.8*	*1.8*	*4.8*	*4.9*	*5.9*	*8.2*
22.	1.25	10	100	8.3	4.4	8.5	8.7	14.5	14.6
23.	1.5	10	100	12.1	7.7	15.0	15.7	24.8	23.7
24.	1.75	10	100	16.7	13.6	20.9	22.3	36.2	33.3
25.	2	10	100	22.1	18.0	28.0	30.3	48.5	46.2
26.	*1.0*	*10*	*200*	*5.1*	*5.1*	*4.8*	*7.7*	*6.6*	*8.2*
27.	1.25	10	200	7.9	9.2	9.9	12.9	15.5	15.5
28.	1.5	10	200	14.5	15.4	16.6	18.7	28.0	25.1
29.	1.75	10	200	20.0	21.4	25.7	27.5	39.4	38.5
30.	2	10	200	27.5	30.6	34.2	38.1	51.7	49.3
31.	*1.0*	*20*	*50*	*5.5*	*1.5*	*4.9*	*2.4*	*3.9*	*6.9*
32.	1.25	20	50	9.2	3.4	8.7	6.9	12.4	15.8
33.	1.5	20	50	13.3	6.2	14.7	15.1	24.1	33.6
34.	1.75	20	50	18.9	12.9	22.5	25.8	38.3	49.6
35.	2.0	20	50	24.0	18.6	27.4	36.7	54.2	66.0
36.	*1.0*	*20*	*100*	*4.8*	*2.8*	*4.2*	*4.9*	*3.9*	*7.7*
37.	1.25	20	100	9.5	6.4	9.0	13.5	14.2	19.7
38.	1.5	20	100	14.4	16.2	16.0	25.6	27.8	37.6
39.	1.75	20	100	20.8	24.8	26.2	40.4	42.0	57.3
40.	2	20	100	27.9	37.3	35.2	53.7	57.3	72.8
41.	*1.0*	*20*	*200*	*4.5*	*5.9*	*5.1*	*8.3*	*5.1*	*7.8*
42.	1.25	20	200	9.3	13.4	9.7	16.6	14.3	20.7
43.	1.5	20	200	16.4	25.7	17.8	31.8	27.5	37.5
44.	1.75	20	200	25.1	41.3	28.8	49.8	44.8	59.7
45.	2	20	200	34.5	55.5	43.6	66.5	61.9	77.4
46.	*1.0*	*40*	*50*	*7.0*	*2.1*	*5.5*	*3.0*	*5.5*	*8.5*
47.	1.25	40	50	13.6	5.1	13.2	11.6	18.6	28.3
48.	1.5	40	50	20.0	14.0	21.5	30.3	31.5	54.1
49.	1.75	40	50	25.5	25.4	27.1	50.4	49.7	79.5
50.	2.0	40	50	32.5	39.3	37.0	66.9	65.8	91.5
51.	*1.0*	*40*	*100*	*5.7*	*3.4*	*3.9*	*7.6*	*4.0*	*6.1*
52.	1.25	40	100	12.2	15.7	10.4	22.7	19.8	31.1
53.	1.5	40	100	19.4	29.7	19.2	48.7	36.4	63.1
54.	1.75	40	100	27.9	50.4	30.1	71.5	54.9	83.8
55.	2	40	100	36.9	69.9	43.4	85.5	72.5	95.2
56.	*1.0*	*40*	*200*	*3.6*	*7.7*	*3.2*	*7.6*	*5.2*	*8.6*
57.	1.25	40	200	9.9	23.8	11.0	27.3	19.7	31.4
58.	1.5	40	200	17.8	46.8	22.7	56.3	36.9	64.6
59.	1.75	40	200	28.1	70.8	37.1	80.3	59.1	86.0
60.	2	40	200	40.8	85.9	52.7	92.0	76.1	96.4

**Table 5:** Power (%) for the ML-NP-EXACT and DerSimonian–Laird inverse-variance techniques heterogeneity τ^2^ = .8

Row #	Odds ratio	Num. of studies	Sample size	Event (disease) probability					
				.005		.01		.05	
				ML-NP-EXACT	DerSimon. Inv.-Var.	ML-NP-EXACT	DerSimon. Inv.-Var.	ML-NP-EXACT	DerSimon. Inv.-Var.
1.	*1.0*	*5*	*50*	*6.1*	*1.0*	*4.6*	*2.6*	*6.9*	*8.7*
2.	1.25	5	50	7.7	1.6	7.0	5.2	10.0	10.8
3.	1.5	5	50	9.0	3.6	9.1	7.9	18.3	17.6
4.	1.75	5	50	10.0	4.6	11.2	9.2	24.2	20.6
5.	2.0	5	50	11.3	5.9	14.4	13.5	29.2	24.2
6.	*1.0*	*5*	*100*	*4.5*	*3.0*	*5.5*	*6.1*	*10.3*	*12.0*
7.	1.25	5	100	7.2	4.9	7.7	9.2	15.0	13.3
8.	1.5	5	100	8.6	7.7	9.9	11.3	22.3	19.1
9.	1.75	5	100	11.7	11.6	16.7	16.8	30.3	24.4
10.	2	5	100	15.3	14.6	18.5	19.8	37.6	29.3
11.	*1.0*	*5*	*200*	*6.4*	*6.2*	*7.2*	*8.4*	*11.8*	*12.2*
12.	1.25	5	200	7.7	9.0	9.7	10.9	19.6	15.1
13.	1.5	5	200	11.6	12.1	16.4	16.1	29.5	20.3
14.	1.75	5	200	14.8	15.8	20.4	20.9	39.0	25.8
15.	2	5	200	19.8	20.9	26.4	25.4	43.9	29.3
16.	*1.0*	*10*	*50*	*6.0*	*1.7*	*5.4*	*3.3*	*7.3*	*9.4*
17.	1.25	10	50	9.5	3.0	9.3	8.0	13.6	14.1
18.	1.5	10	50	12.3	5.5	13.1	12.8	22.8	23.1
19.	1.75	10	50	15.6	9.4	18.0	17.8	33.5	33.0
20.	2.0	10	50	18.4	12.8	22.1	22.3	41.1	41.2
21.	*1.0*	*10*	*100*	*5.1*	*4.1*	*5.7*	*7.0*	*6.9*	*8.8*
22.	1.25	10	100	8.6	7.8	11.3	12.9	16.3	14.5
23.	1.5	10	100	13.3	12.5	16.5	19.1	26.9	25.6
24.	1.75	10	100	17.1	17.7	22.7	27.3	38.6	36.6
25.	2	10	100	22.3	25.4	30.1	34.8	50.2	46.9
26.	*1.0*	*10*	*200*	*5.6*	*7.0*	*5.3*	*8.5*	*8.1*	*9.5*
27.	1.25	10	200	10.6	12.9	12.2	14.5	18.2	14.8
28.	1.5	10	200	16.3	20.3	21.1	24.2	28.7	25.2
29.	1.75	10	200	20.9	25.7	26.8	28.7	42.7	37.1
30.	2	10	200	30.6	36.0	37.7	40.3	49.2	44.8
31.	*1.0*	*20*	*50*	*6.0*	*3.5*	*5.0*	*5.8*	*4.4*	*9.6*
32.	1.25	20	50	8.9	6.1	10.0	14.4	13.8	22.5
33.	1.5	20	50	13.6	11.6	15.8	23.2	24.7	36.9
34.	1.75	20	50	19.3	19.0	23.9	34.5	40.2	53.2
35.	2.0	20	50	25.8	27.4	31.3	45.6	50.5	66.0
36.	*1.0*	*20*	*100*	*5.0*	*5.6*	*4.0*	*8.5*	*4.8*	*8.5*
37.	1.25	20	100	9.9	12.8	11.1	20.5	12.0	21.3
38.	1.5	20	100	15.4	23.2	18.2	34.3	27.3	38.6
39.	1.75	20	100	21.8	35.9	25.0	45.9	37.9	54.6
40.	2	20	100	30.0	46.9	38.2	60.1	56.2	71.2
41.	*1.0*	*20*	*200*	*4.0*	*9.1*	*4.8*	*9.7*	*5.1*	*8.8*
42.	1.25	20	200	8.9	19.8	10.2	21.5	15.4	21.6
43.	1.5	20	200	16.8	33.5	21.3	37.8	27.8	38.2
44.	1.75	20	200	27.0	48.3	34.7	53.2	43.8	56.6
45.	2	20	200	36.8	60.4	44.9	66.9	59.2	72.1
46.	*1.0*	*40*	*50*	*6.6*	*3.3*	*5.5*	*9.6*	*4.4*	*11.4*
47.	1.25	40	50	12.9	12.5	11.3	25.0	13.4	33.1
48.	1.5	40	50	17.9	25.0	18.4	44.5	29.7	65.1
49.	1.75	40	50	25.8	39.7	27.6	67.1	45.1	80.2
50.	2.0	40	50	30.9	55.2	37.6	78.4	61.7	93.4
51.	*1.0*	*40*	*100*	*5.2*	*9.7*	*3.7*	*14.9*	*4.1*	*10.5*
52.	1.25	40	100	10.6	25.3	8.5	36.7	15.7	37.1
53.	1.5	40	100	17.8	45.9	18.1	57.3	32.0	65.5
54.	1.75	40	100	25.8	64.8	30.0	76.1	51.7	83.5
55.	2	40	100	34.5	77.9	45.4	87.8	70.8	95.2
56.	*1.0*	*40*	*200*	*2.6*	*14.6*	*3.4*	*13.0*	*3.6*	*10.3*
57.	1.25	40	200	7.8	37.1	11.5	37.8	13.3	32.3
58.	1.5	40	200	17.2	59.7	26.5	61.9	32.8	61.7
59.	1.75	40	200	31.6	77.0	41.5	82.2	53.8	85.2
60.	2	40	200	46.2	89.3	57.4	93.1	74.0	94.6

There are three general findings from the direct comparison of the ML-NP-EXACT technique with the DL technique.

**Fig. 5:**
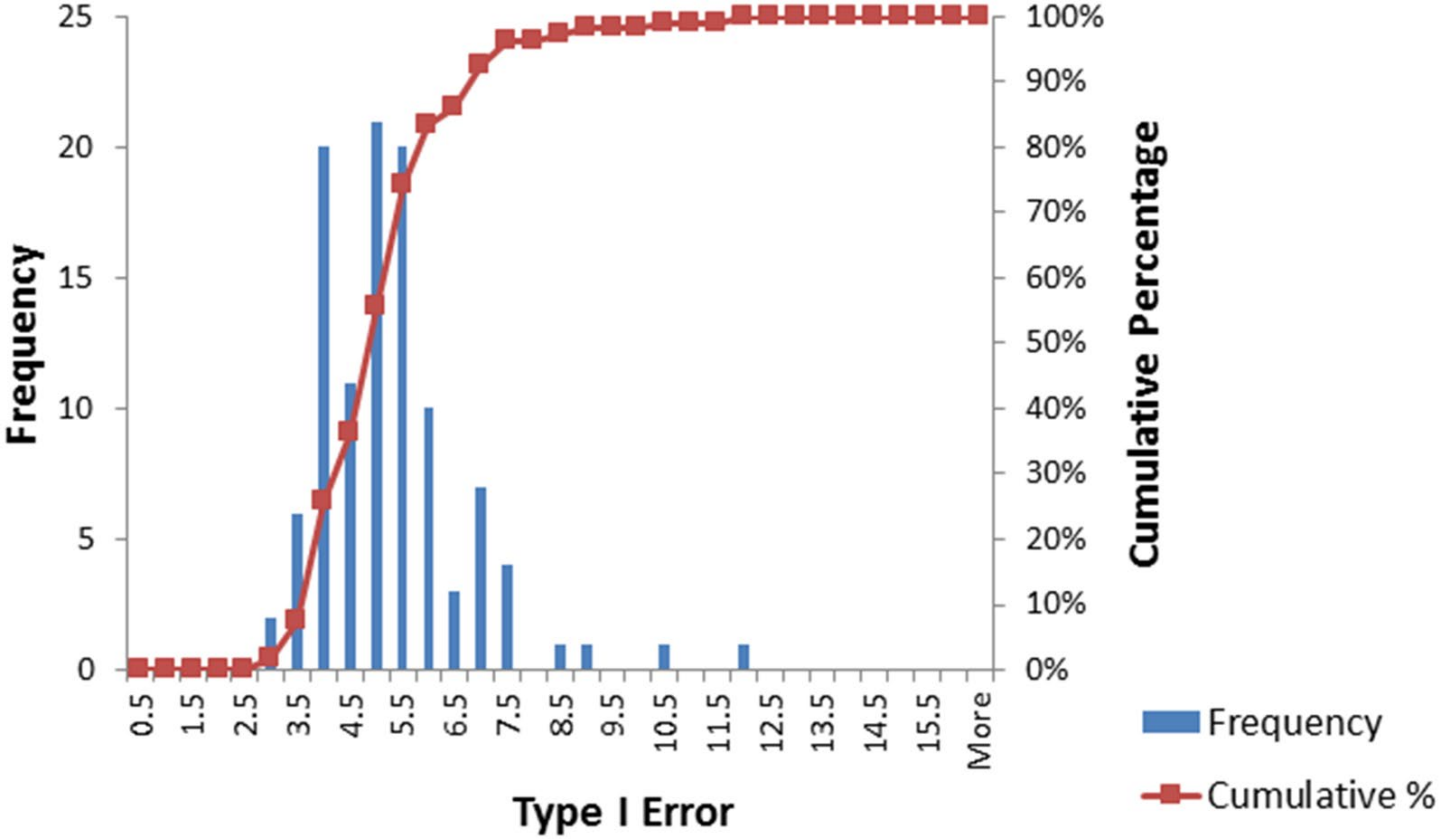
Histogram of the Type I Error for the ML-NP-EXACT technique

1. For the homogeneous case of τ^2^ = 0, the ML-NP-EXACT technique yielded a Type I Error value centered on the pre-specified level of .05 for practically all combinations of event probability, number of contributing studies and sample size as show in Table 3. However, as shown in Table 3, the DL technique consistently returned a Type I Error value well below .05 and correspondingly low levels of power for Odds Ratios greater than 1.0. In order to compare the power for Odds Ratios > 1.0, an upper limit on Type I Error needs to be established. Using a Type I Error level of 7.5%, the ML-NP-EXACT technique demonstrated a larger power in over 73% of the comparisons.
2. For the most heterogeneous τ^2^ = .8 case and to a lesser degree for τ^2^ = .4, the DL technique frequently failed to protect the pre-specified Type I Error value of .05 while the ML-NP-EXACT technique was superior. Figures 5 and 6 are the histograms of the 108 Odds Ratio = 1.0 data points for the ML-NP-EXACT and DL techniques respectively. The ML-NP-EXACT Type I Error was centered approximately on the nominal .05 value. The DL technique shows concentrations at very small values of Type I Error and a very wide range of higher values of Type I Error. Using the arbitrary Type I Error limit of 7.5%, there was ten times the number of violations of this level for the DL technique (40) compared to the ML-NP-EXACT technique (4).

**Fig. 6:**
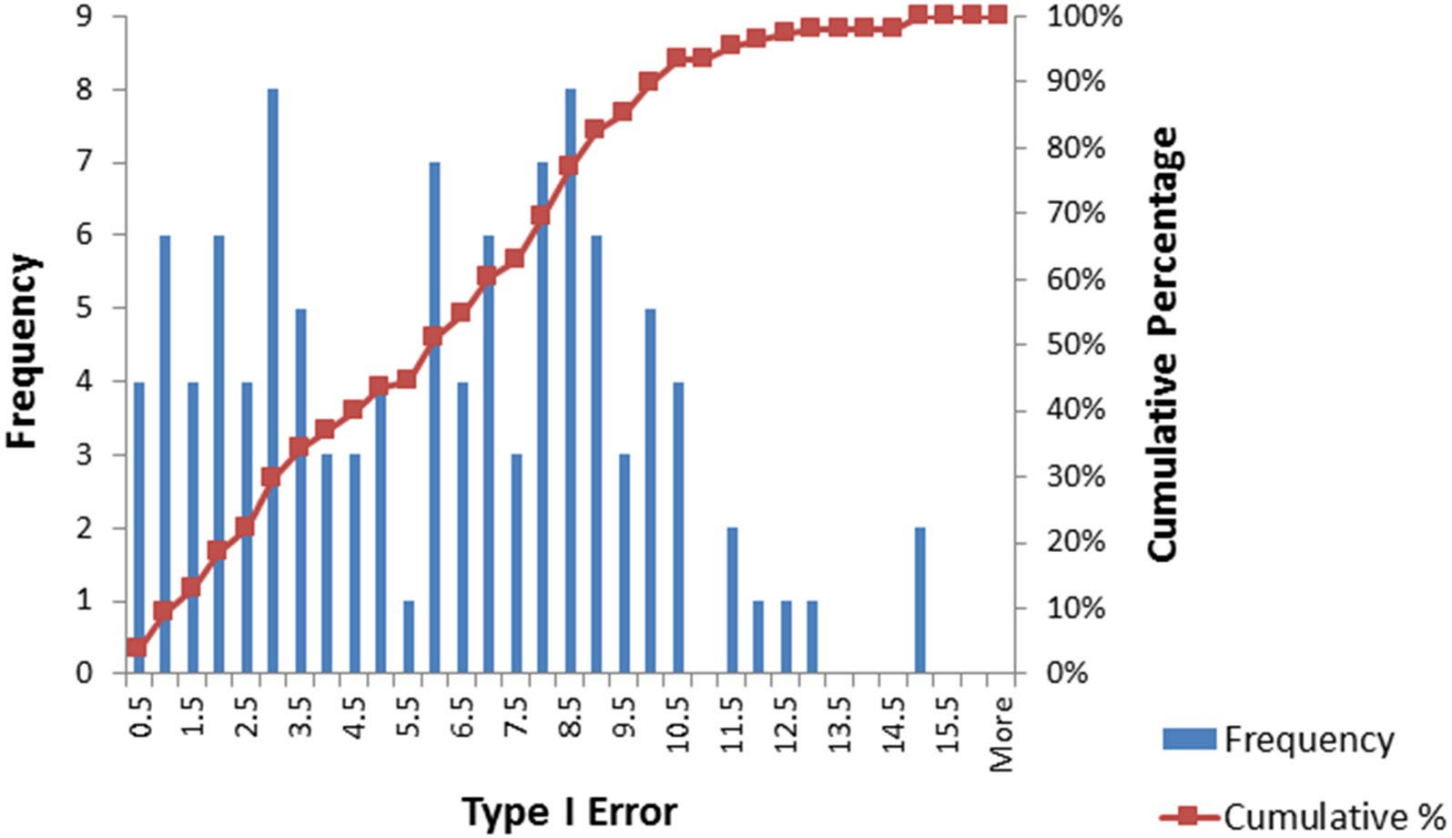
Histogram of the Type I Error for the DerSimonian–Laird inverse-variance technique
  This inability of the inverse-variance DerSimonian–Laird technique to protect a pre-specified value of Type I Error has been shown in previous studies (See, for example [15, 25]). In the Bhaumik et al. study [15], Fig. 2a shows Type I Error rates up to approximately 15% for the range of µ of -3 to -5 of primary interest in the current study. This same level of violation of the pre-specified level of Type I Error was found in the present simulations.
  Finally, three of the four occasions when Type I Error for the ML-NP-EXACT technique exceeded the 7.5% level had just five contributing studies. As discussed earlier, the probability for a single study to incorrectly skew the overall test statistic towards statistical significance increases sharply as the number of contributing studies decreases.
3. As expected, when the DL technique protected the Type I Error, it tended to be more powerful than the ML-NP-EXACT technique for higher values of odds ratio, event probability, number of contributing studies and sample size. These higher values lead to less sparse contingency tables and the increased appropriateness of a large sample asymptotic method such as the DL Inverse Variance technique.

As shown clearly in Tables 3, 4 and 5, the two variables under the experimenter’s direct control, Number of Studies and Sample Size, both had powerful effects on the power for the ML-NP-EXACT technique. Somewhat surprisingly, the heterogeneity variable, τ^2^, did not largely affect power. However, its inflationary influence on Type I Error needed to first be “tamed.”

### Extending the ML-NP-EXACT technique

#### Unbalanced designs

Additional Monte Carlo testing was done for unbalanced designs (unequal sample sizes in the exposure and no exposure arms of the contributing studies) and meta-analyses with unequal sample sizes across contributing studies. Table 6 shows the sample sizes for the two groups for a typical unbalanced design in which the control sample size is twice the exposure group sample size. The sum of the two sample sizes across the two arms of each study was chosen to be 200. This design allows direct comparison of the simulation results with the balanced designs of Tables 3, 4 and 5.

**Table 6:** Sample sizes for simulation of unbalanced designs number of studies = 10

Group	Study #									
	1	2	3	4	5	6	7	8	9	10
Exposure	66	66	66	66	66	66	66	66	66	66
Control	134	134	134	134	134	134	134	134	134	134

Table 7 shows the results of the simulation for a heterogeneity value τ^2^ = 0, Event (“disease”) Probability of .05, and Sample Size (per arm) of 100, at the same five levels of Odds Ratio used above. The simulation run consisted of 2000 replications as in Tables 3, 4 and 5.

**Table 7:** Power (%) for the unbalanced design of Table 6 for the ML-NP-EXACT and DerSimonian–Laird inverse-variance techniques heterogeneity τ^2^ = 0; event probability = .05; number of studies = 10; sample size across both arms = 200

Technique	Odds ratio				
	1.0	1.25	1.5	1.75	2.0
ML-NP-EXACT	4	12.1	23. 5	37.9	50.7
DerSimonian–Laird inv. variance	7.3	16.1	25.7	41.9	54.9

As the results in Table 7 indicate, the ML-NP-EXACT power in the unbalanced scenario is slightly smaller for all values of Odds Ratio relative to the DerSimonian–Laird method. Type I Error (Odds Ratio = 1.0) was close to the prespecified value of five percent for the ML-NP-EXACT technique and was reasonably close to five percent for the DL technique.

#### Unequal sample size designs

Table 8 shows the sample sizes for the exposure and control groups for each of the contributing studies for a design with unequal sample size **across** the contributing studies. This particular design was chosen as a relatively extreme case. As can be seen, the average sample size for both the exposure and control groups was maintained at 100 to allow comparison of the simulation results with the equal sample size scenarios of Tables 3, 4 and 5.

**Table 8:** Sample sizes for simulation of unequal sample size designs number of studies = 10

Group	Study #									
	1	2	3	4	5	6	7	8	9	10
Exposure	175	25	175	25	175	25	175	25	175	25
Control	175	25	175	25	175	25	175	25	175	25

Table 9 shows the results of the simulation for a heterogeneity value τ^2^ = 0, Event (“disease”) Probability of .05, and Average Sample Size (across both study arms) = 200, at the same five levels of Odds Ratio as used above. The simulation run consisted of 2000 replications as in Tables 3, 4 and 5.

**Table 9:** Power (%) for the unequal sample size design of Table 8 for the ML-NP-EXACT and DerSimonian–Laird inverse-variance techniques heterogeneity τ^2^ = 0; event probability = .05; number of studies = 10; average sample size (across both study arms) = 200

Technique	Odds ratio				
	1.0	1.25	1.5	1.75	2.0
ML-NP-EXACT	6.2	12.1	24.0	35.5	47.0
DerSimonian–Laird inv. variance	9.5	13.9	23.2	35.1	44.4

The ML-NP-EXACT power in this unequal sample size condition was slightly superior to the DerSimonian–Laird technique. More importantly, the ML-NP-EXACT was superior at protecting the pre-specified level of Type I Error.

## Discussion and conclusions

This research has developed an exact test for the meta-analysis of dichotomous categorical data. The ML-NP-EXACT technique was strongly superior to the DerSimonian–Laird technique in maintaining a pre-specified level of Type I Error. As shown, the DerSimonian–Laird technique demonstrated many large violations of this level. Given the various biases towards finding statistical significance prevalent in epidemiology today, a strong focus on maintaining a pre-specified level of Type I Error would seem critical (see, e.g., [30]). In addition, for the homogeneous case, the new technique is generally more powerful than the typically used large sample asymptotic DerSimonian–Laird inverse-variance technique for realistic, smaller values of disease probability across a large range of odds ratios, number of contributing studies, and sample size. While statistical programs providing exact solutions already exist such as Cytel’s StatXact, they are beyond the means of most practicing statisticians and epidemiologists.

The technique developed here is written in the almost universal statistical language of R and is freely available from the author. As such, it is hoped that other researchers will be able to extend and improve this initial version.

As outlined in this report, the use of meta-analysis in epidemiology is increasing very rapidly and appears to be meeting an important need. However, fortunately, inexpensive and readily available computer power has also increased vastly in the past forty years. For example, task speed as measured in Million Instructions per Second (“MIPS”) has increased from a fraction of a MIP for the IBM 370 mainframe computer in 1972 to thousands of MIPS for an Intel Pentium processor personal computer today [31]. By using the techniques developed here and the computer power available to all researchers today, unnecessary sources of error can be readily eliminated.
